# Green-Hydrogen Energy Share: An Intuitive Metric to Compare Energy Density and H_2_ Efficiency in Biofuels and E‑Fuels

**DOI:** 10.1021/acsomega.5c09592

**Published:** 2026-03-27

**Authors:** Ada Robinson Medici, Aristide Giuliano, Nicola Pierro, Isabella de Bari, Stavros Papadokonstantakis

**Affiliations:** a Institute of Chemical, Environmental and Bioscience Engineering, 27259 TU Wien,Wien 1060, Austria; b ENEA, Italian National Agency for New Technologies, Energy and Sustainable Economic Development, S.S. 106 Ionica, km 419 + 500, Rotondella, MT 75026, Italy

## Abstract

This communication proposes two indices to quantify hydrogen utilization efficiency and compare biofuels and e-fuels on a common energy basis: Hydrogen-Energy Share coefficient, HES [MJ_H2_/kg_fuel_], and its complementary, Hydrogen Energy fraction, HES% [MJ_H2_/MJ_fuel_]. Mass- and energy-balanced data sets are normalized to 1 MJ of produced-fuel (lower heating value). External-energy demand is disaggregated into feedstock provision, synthesis-plant operation, and renewable power for green hydrogen (G-H2) electrolysis. Then, the metrics are applied across 11 industrially relevant pathways, 5 e-fuels, and 6 biomass-to-liquids biofuels, as a compact demonstration data set. The analysis is restricted to liquid fuel routes; hydrogen storage vectors (e.g., ammonia and LOHCs) are outside the present scope. Total energy input spans an order of magnitude, from 0.19 MJ_H2_/MJ_HVO_ to 2.62 MJ_H2_/MJ_e‑FT_. In e-fuel pathways, most input is the electricity used to produce G-H_2_ (1.6–2.0 MJ_H2_/MJ_e‑fuel_) with HES up to 60 MJ_H2_/kg_e‑CH4_ and HES% ≥ 100%. Bioroutes use little electrolytic hydrogen but depend on sustainable biomass *y* (0.08–1.06 MJ_biomass_/MJ_biofuel_); HES ranges 3–38 MJ_H2_/kg_biofuel_ and HES% ≤ 80%. Defined purely from energy balances, HES/HES% are proposed as first-order hydrogen-energy metrics to be used alongside, rather than instead of, detailed techno-economic and environmental assessments. These indexes make the electricity-versus-biomass trade-off explicit and intuitive in the deployment discussion: bioroutes where low-carbon power is scarce but biomass is available, electrofuels where cheap clean power and concentrated CO_2_ are colocated.

## Graphical abstract

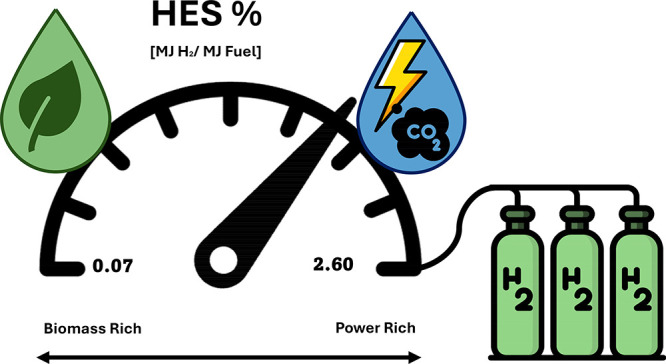

## 1 Introduction

The growing integration of green-hydrogen (G-H_2_) produced from renewable sources in the global energy system underscores the key role that hydrogen can play in decarbonizing multiple industries. Hydrogen’s flexibility is attractive in numerous processes, including the production of e-fuels (fuels produced from CO_2_ and green H_2_) and biofuels (fuels produced from a biogenic carbon source plus G-H_2_).^1^ Electrolysis can also buffer the variability of wind and solar generation from Variable Renewable Energy Sources (VRES). Nevertheless, hydrogen’s cryogenic boiling point (−252.8 °C) and low volumetric energy density make long-distance storage and transport technically demanding compared to other fuels.^2^

To address this issue, the concept of Power-to-X (PtX) has emerged, which involves the conversion surplus renewable electricity, typically via electrolytic G-H_2_, into various forms called “X”. This approach is increasingly favored by G-H_2_ producers, as it provides a more viable solution to overcome storage and transportation challenges.^3^ Alternatively, hydrogen can be a reagent in many processes for the production of fuels and chemicals, and the resulting synthetic and biogenic fuels also participate in complementary X-to-Power chains, where they are reconverted to electricity in fuel-cell-based cogeneration, including integrated systems with biomass gasifiers or anaerobic digesters.^4,5^

E-fuels are synthetic fuels made exclusively from renewable hydrogen and a carbon source that is fossil-free or fully captured and reused (e.g., CO_2_ from industrial flue gas, biogenic CO_2_, or direct air capture). Biofuels derive their carbon primarily from biomass (e.g., lipids, lignocellulose, sugars) and use additional green hydrogen to either upgrade or fully hydrogenate the feedstock.^6^ Among sustainable fuels, drop-in fuels offer the advantage that they are functionally equivalent to fossil-based fuels and compatible with existing engines, so they can be used without modifications to infrastructure, within the blending limits set by current fuel standards. They include hydroprocessed esters and fatty acids (HEFA), hydrotreated vegetable oils (HVO), Fischer–Tropsch fuels (FT), and synthetic paraffinic kerosenes (SPK) whose synthesis requires a hydrotreatment step.^7^ The use of G-H_2_ in biorefineries would have the advantage of allowing local integration while avoiding costs and environmental impacts due to long-term storage and transportation. Locally pairing G-H_2_ electrolyzers with biorefineries can exploit the shared geographic dispersion of renewable electricity and biomass while avoiding the costs and impacts of long-distance H_2_ transport.

An interesting analysis can be conducted to identify the differences in energy content among this wide range of alternative fuels by focusing on their G-H_2_ energy share. However, choosing between biofuels, limited by a sustainable biomass supply, and e-fuels, which are much more electricity-intensive, is not straightforward. Three, tightly linked questions are therefore asked: How many MJ of electrolytic H_2_ must be invested per kilogram of fuel? What fraction of the fuel’s own heating value comes from that hydrogen? For which end-use sectors is such an investment justified? There is an ongoing debate about sustainable fuels and the opportunity they offer to decarbonize the transport.^8,9^ Recent techno-economic, life-cycle, and optimization studies address these questions for specific applications, for example, heavy-duty road transport,^10^ automotive e-fuels,^11^ shipping fuels,^12^ and integrated biomass–G-H_2_ e-fuel systems.^13^ They report detailed mass- and energy-balance data together with route-specific performance indicators. However, to our knowledge, there is currently no straightforward way to compare e-fuels and hydrogenated biofuels on the same hydrogen-intensity basis: existing studies do not provide a simple number, expressed per unit of fuel energy, that can be directly derived from their reported data. Because the proposed indices depend only on the specific hydrogen demand and the fuel lower heating value, they can be calculated consistently from published mass- and energy-balance data, even when modeling assumptions and system boundaries differ. HES and HES% thus provide a single measure of how much fuel energy is obtained per unit of G-H_2_ input, offering a clear, energy-based decision tool for the preliminary allocation of scarce green hydrogen, to be used alongside, rather than instead of, detailed techno-economic or environmental assessments. To carry out this comparison, the analysis is restricted to e-fuels and hydrogen-assisted biofuels pathways as final energy carriers; hydrogen storage technologies and hydrogen carrier concepts (such as liquid hydrogen, ammonia, and liquid organic hydrogen carriers) are outside the system boundaries considered here. Within this scope, the three-step framework is applied (Figure 1).

**Figure 1:**
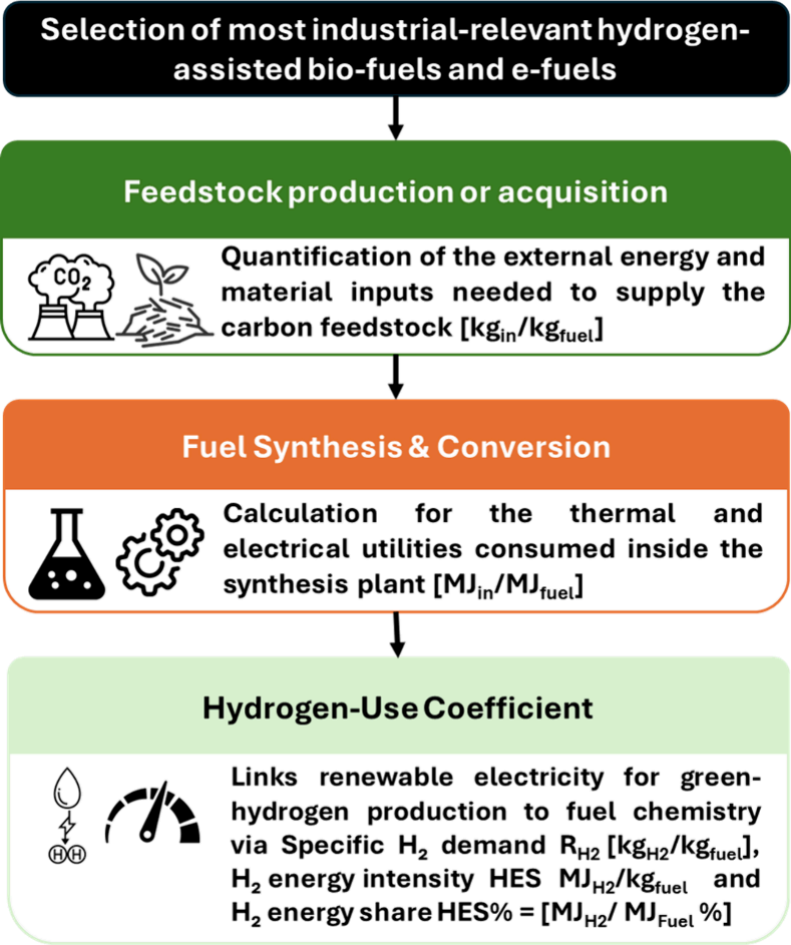
Analytical framework of this work. Selection of the most industrially relevant hydrogen-assisted biofuels and e-fuels. Compilation of complete mass and energy balance databases for every pathway, from feedstock to finished fuel. Quantification of the external energy and material inputs needed to supply the carbon feedstock (green) (i.e., captured CO_2_ or biomass; water used in electrolysis is not counted as a feedstock input); calculation for the thermal and electrical utilities consumed inside the synthesis plant (orange); three metrics: Specific H_2_ demand R_H2_ [kg_H2_/kg_fuel_], H_2_ energy share HES = LHV_H2_*R_H2_ [MJ_H2_/kg_fuel_], and H_2_ energy fraction HES% = HES/LHV_Fuel_ [%] link renewable electricity for green-hydrogen production to fuel chemistry (light green). The inputs are normalized to MJ_in_/MJ_fuel_ and kg_in_/kg_fuel_.

## 2 Methodology

### 2.1 Analytical Framework

To evaluate each fuel pathway, the lifecycle stages considered are (1) feedstock production or acquisition (e.g., CO_2_ capture or biomass cultivation/collection), (2) fuel synthesis/conversion, and (3) green hydrogen supply. For each stage, thermal energy and electrical energy inputs required per unit of fuel produced are tracked.^14^

### 2.2 Definition of Pathways

From a thermal point of view, hydrocarbon synthesis processes through the conversion of CO_2_ and H_2_ are always self-sufficient, as the reactions involved are mostly exothermic, which means that the production of e-fuel essentially requires electrical energy for hydrogen production. All synthesis steps are treated as ideal-gas reactions obeying standard equilibrium relations $K_{p}\left(T\right)=\prod_{i}{\left(\frac{p_{i}}{p^{◦}}\right)}^{\nu_{i}}$ with rates driven by reactant partial pressures; with *K*_*p*_(*T*) obtained from the standard Gibbs free energy of reaction, *K*_*p*_ = exp [−Δ*G*_*R*_^°^/(*RT*)], and its temperature dependence governed by the van’t Hoff relation, such exothermic hydrogenation reactions are favored at elevated pressure and relatively low temperature. Reactions follow the standard enthalpy of reaction, $\Delta H_{R}^{{}^{\circ}}=\sum_{i}\nu_{i}\Delta H_{f,i}^{{}^{\circ}}$ , where ν_*i*_ are the stoichiometric coefficients and Δ*H*_*f*, *i*_^°^ are the standard enthalpies of formation of the participating species. The higher the H/C ratio in the fuel, the higher the quantity of hydrogen needed to deoxygenate the CO_2_ with the production of water and heat:^15^

$$nCO_{2}+\left(3n+1\right)H_{2}⇌C_{n}H_{2n+2}+2nH_{2}O$$ 1

where the stoichiometric coefficient value of hydrogen to that of CO_2_ ( $\frac{3n+1}{n}$ ) varies between 3 and 4 depending on *n*. In particular, for the synthesis of methane, the hydrogen to carbon ratio (H/C) is equal to 4, and as the carbon chain length increases, it tends to 3.^16^ The stoichiometric coefficients can vary depending on the nature of the fuel, whether they are linear or isomerized alkanes. In the case of e-fuels containing oxygen (e.g., methanol and dimethyl ether), the stoichiometric coefficient of hydrogen to CO_2_ is equal to 3.^17^

A second category of e-fuels is represented by oxymethylene ether (OMEx), having the advantages of containing oxygen between each C–C bond with a consequent lower H_2_ consumption and a better combustion process and NOx emissions:^18^

$$nCO_{2}+\left(2n+2\right)H_{2}⇌{CH}_{3}O-{\left({CH}_{2}O\right)}_{n-2}-{CH}_{3}+\left(n+1\right)H_{2}O$$ 2

On the other hand, the LHV of OMEx is significantly lower than that of FT-e-fuels (about 20 MJ/kg vs 40 MJ/kg).

The process of synthesizing FT fuels and methanol/DME is also well-established, conventionally using conditioned syngas to reach a H_2_/(CO + CO_2_) synthesis ratio of around 1.5–2.5.^22,23^ The conditioning processes have been extensively studied and include WGS steps to increase the H_2_ and CO_2_ content, followed by CO_2_ capture. An alternative method involves increasing the H_2_ content by adding it directly to the syngas mixture rich in CO and CO_2_. Typically, the H_2_/CO/CO_2_ ratios vary in the ranges of 0.5–1.5/0.5–2/0.5–2, and high quantities of hydrogen must be added to achieve a syngas composition suitable for the synthesis of hydrocarbons (C1-biomethane/Cn-FTdiesel) or methanol/DME. In this case, the entire quantity of carbon in the raw material (biomass or waste) is converted into products, requiring pure hydrogen. In this study, a representative syngas composition was considered: H_2_/CO/CO_2_ → 1/1/1 (synthesis ratio before hydrogen addition = 0).

A similar analysis can be performed to produce hydrogenated biofuels, for which the synthesis can be carried out starting from different types of renewable carbon sources: lipids (triglycerides) or fatty acids, syngas, and ethanol. The most common and currently industrialized process in several countries is represented by the hydrogenation of triglycerides and fatty acids, producing HEFA, or HVO. The compositions of these fuels are very similar to those of naphtha, diesel, and kerosene obtained from fossil sources. In addition to the heavier “liquid” hydrocarbons, short-chain alkanes (C2–C4) similar to liquefied petroleum gas (LPG) with a high propane content are obtained as coproducts.^24^

$$C_{n}H_{2n+1}COOH+3H_{2}⇌C_{n+1}H_{2n+4}+2H_{2}O$$ 3

The last category of biobased intermediates for the synthesis of alternative fuels is represented by fermentation-derived ethanol, which can be converted into diesel and/or aviation fuels through conversion steps of dehydration, oligomerization, and hydrogenation:

$$\frac{n}{2}C_{2}H_{6}O+H_{2}⇌C_{n}H_{2n+2}+\frac{n}{2}H_{2}O$$ 4

The stoichiometric reaction in this case is always balanced with the addition of only one hydrogen molecule for each ethanol molecule converted into alkanes. This makes the process particularly interesting for the production of hydrocarbons from biomass sources without the need to use large amounts of hydrogen and no need for CO_2_ capture or storage (unlike FT, SAF, MeOH, or DME).^25–28^

Table 1 compares the main synthetic pathways for both e-fuels and biobased fuels, including typical feedstocks, chemical reactions, mass yields, and technology readiness. All of the synthesis reactions listed here are reversible because product formation is thermodynamically governed. Following Le Chatelier’s principle, these strongly exothermic syntheses, such as the Sabatier reaction, are favored by high pressure and relatively low temperature, while industrial operation employs moderate temperatures and catalysts to balance equilibrium constraints with sufficient reaction rates.

**Table 1:** Representative E-Fuel and Biofuel Pathways, with Feedstocks, Synthesis Routes, Indicative Mass Yields (Kilograms of Feedstock per Kilogram of Fuel), and the Technology Readiness Level (TRL)

e-fuel	synthesis pathway	yield [kg_in_/kg_fuel_]	TRL	ref.
hydrogen (H_2_)	water electrolysis using renewable power: 2H_2_O ⇌ 2H_2_ + O_2_	0.66	9	^19^
e-methane (CH_4_)	Sabatier methanation: CO_2_ + 4H_2_ ⇌ CH_4_ + 2H_2_O	0.36	6–7	^19^
e-methanol (CH_3_OH)	hydrogenation of CO_2_ to methanol: CO_2_ + 3H_2_ ⇌ CH_3_OH + H_2_O (Cu/Zn)	0.73	7–8	^19^
e-dimethyl ether (DME)	from e-methanol dehydration: 2CO_2_ + 6H_2_ ⇌ CH_3_OCH_3_ + 3H_2_O or direct CO/CO_2_ + H_2_ conversion (catalytic)	0.52	6–7	^20^
e-Fischer–Tropsch hydrocarbons (FT)	reverse WGS to form CO, then CO + H_2_ → synthetic hydrocarbons: *n*CO_2_ + (3*n* + 1)H_2_ ⇌ C_*n*_H_2__*n*__+__2_ + 2*n*H_2_O (*n* ≈ 10)	0.7	5–6	^19^
e-oxymethylene ethers (OMEx) *x = 3–5	stepwise formation from methanol/formaldehyde derived via CO_2_ + H_2_: 4CO_2_ + 10H_2_ ⇌ CH_3_O(CH_2_O)_3_CH_3_ + 7H_2_O	0.4–0.6	4–5	^18^

**biofuel**	**synthesis pathway**	**yield**	**TRL**	**ref.**
biomass-to-liquid (BTL FT-Biofuels)	biomass gasification → syngas → Fischer–Tropsch → syncrude → diesel/jet. H_2_/CO/CO_2_ = 1/1/1	0.25–0.4	6–7	^21^
bio-methane	from syngas H_2_/CO/CO_2_ = 1/1/1	0.4		^21^
bio-DME	from syngas (biomass gasification) H_2_/CO/CO_2_ = 1/1/1	0.35–0.5	6–7	^21^
bio-methanol	gasification of biomass to syngas, then catalytic methanol synthesis H_2_/CO/CO_2_ = 1/1/1	0.4–0.55	7–8	^21^
HVO/HEFA	hydrotreating vegetable oils/fats with H_2_ → paraffinic diesel/jet	0.75–0.85	9	^21^
biojet/sustainable aviation fuel (SAF)	includes HEFA-SPK, ATJ, SIP (various routes to jet-range molecules). GD/SAF from EtOH	0.7–0.8	6–9	^21^

### 2.3 Construction of Energy Input Tables

Energy input and output tables are compiled for various fuel production pathways by accounting for each stage of the process: (1) green hydrogen production, (2) carbon feedstock sourcing (CO_2_ capture or biomass provision), and (3) fuel synthesis and upgrading. This three-stage structure is generic and can be refined or extended (e.g., alternative H_2_ routes, different CO_2_ sources, additional feedstocks such as water) whenever consistent, pathway-specific data are available. In the present comparison, we use this minimal representation for illustration. Below, we detail the assumptions and data sources for each stage. All energy quantities are expressed per unit of fuel produced (e.g., MJ per MJ of fuel LHV, or MJ per kg fuel) to allow comparison of overall conversion efficiency. Key data are drawn from current literature and industrial benchmarks, with full tabulated values provided in the Supporting Information.

#### 2.3.1.1 Green Hydrogen Production

A critical variable in the synthesis of e-fuels is the origin of hydrogen used in these pathways. In this study, hydrogen is assumed to be exclusively green hydrogen, produced via water electrolysis powered by 100% renewable electricity (e.g., from wind, solar PV, or hydroelectric sources). An LHV efficiency of 60% is adopted, so that splitting water requires 50–55 kWh/kg H_2_ (180–200 MJ/kg including balance-of-plant losses).^29^ By isolating the contribution of hydrogen and CO_2_ origins (see [Sec sec2.3.2] Carbon Feedstock Sourcing section below) in this analysis, it becomes possible to define a clear hierarchy of renewable fuel pathways based on their material and energy efficiency, enabling optimized decisions for decarbonized fuel deployment. Details about the renewable-powered water-electrolysis technologies are provided in the Supporting Information (S2).^30–36^

#### 2.3.1.2 Hydrogen Coefficient

Renewable fuels can be scored also based on the amount of hydrogen needed to obtain them and, consequently, also concerning the chemical energy represented by the hydrogen that is deposited in the fuel.^37^ To this scope, hydrogen-use coefficients have been calculated as

$$R_{H2}=\frac{\mathrm{mass}\;\mathrm{of}\;\mathrm{hydrogen}}{\mathrm{mass}\;\mathrm{of}\;\mathrm{fuel}}$$ 5

$$\mathrm{HES}={\mathrm{LHV}}_{H2}\times R_{H2}$$ 6

$$\mathrm{HES}\%=\frac{\mathrm{HES}}{{\mathrm{LHV}}_{\mathrm{fuel}}}\times 100\%$$ 7

where LHV_H2_ = 120 MJ/kg is the calorific value of hydrogen, LHV_fuel_ (MJ_fuel_/kg_fuel_) is the calorific value of the renewable fuel obtained, HES is the Hydrogen-energy share coefficient, HES% is the Hydrogen energy fraction, and *R*_H2_ is the amount (kg_H2_/kg_fuel_) of hydrogen needed to produce 1 kg of fuel. Based on this definition, pure hydrogen corresponds to *R* = 1, and HES% = 100%. All e-fuels have an LHV represented by the amount of hydrogen needed to produce them, with e-methane at the forefront. For e-methane applying the hydrogen metrics, the value reflects the ratio between the calorific value of the hydrogen used and that of the fuel produced (i.e., energy in, energy out).^9^

In this case, the specific hydrogen demand *R*_H2_ is 0.5 kg_H2_/kg_CH4_, giving a hydrogen-energy intensity HES of 60 MJ_H2_/kg_CH4_. This corresponds to a hydrogen-energy share HES% of 120%, meaning about 60% of the LHV of methane results from the energy contained in the hydrogen used during its synthesis, when produced from syngas with a H_2_/CO/CO_2_ ratio of 1/1/0. Among biofuels, ethanol-derived SAF shows that *R*_H2_ is 0.014 kg_H2_/kg_fuel_, yielding an HES of 1.7 MJ_H2_/kg_fuel_ and an HES% of 4%, so less than 4% of the drop-in fuel’s energy originates from added green hydrogen. The proposed HES/HES% framework is general and can be combined with different definitions of the hydrogen coefficient (e.g., net hydrogen stored in the fuel, or total hydrogen fed including recycle/purge losses), provided that these are clearly defined. In this Letter, a net, fuel-centric definition of *R*_H2_ is adopted because it can be derived consistently from published mass balances for all pathways considered.

#### 2.3.2 Carbon Feedstock Sourcing

Equally important are the origin and quality of the CO_2_ feedstock. CO_2_ can be captured from a range of sources, each with distinct thermodynamic, energetic, and environmental implications. The most used industrial sources include flue gases from fossil fuel combustion in power plants, cement kilns, and steel manufacturing facilities. These streams typically contain 5–20% CO_2_ by volume and require postcombustion capture technologies such as amine scrubbing, which add significant thermal energy demand (typically 2.5–3.5 GJ/ton CO_2_).^38^ In the context of e-fuel synthesis, these upstream differences in CO_2_ sourcing must be carefully considered as they significantly affect the overall system efficiency and environmental footprint. A detailed summary about various CO_2_ capture routes, each with their own energy footprints, is provided in the Supporting Information (S3).^39,41–52^ In practice, many e-fuel projects today leverage industrial CO_2_ waste streams (e.g., from fertilizer or ethanol plants) as a convenient source, which requires minimal energy to collect, avoiding the full DAC energy penalty in the near term. However, since we prefer to model an unconstrained availability of CO_2_, this communication assumes the energy demand of a commercial scale amine scrubbing plant.^40^

Assuming drop-in biofuel production begins with biomass feedstock, which can range from energy crops (corn, sugar cane, oilseeds) to lignocellulosic residues (wood chips, straw).^53^ Unlike CO_2_, biomass inherently contains chemical energy (stored solar energy). The energy balance distinguishes between feedstock intrinsic energy (the LHV of the biomass, which ultimately contributes to fuel energy) and external energy inputs (any additional heat, power, or reagents) required to condition the biomass for conversion. Triglyceride-rich oils are advantageous: they have a higher LHV (about 30 MJ/kg) and require only minimal pretreatment before fuel synthesis. By contrast, lignocellulosic biomass has a lower LHV (15–20 MJ/kg) and a heterogeneous composition (lignin, cellulose, hemicellulose, ash, moisture), so it demands energy-intensive conditioning (e.g., high oxygen demand during gasification or high-temperature steam/chemical steps in second-generation sugar platforms).^54^

#### 2.3.3 Fuel Synthesis and Upgrading

The final stage is the actual conversion of hydrogen and carbon feeds into the target fuel. This can involve reactors (with heating or cooling needs), separation or purification steps, and compression or pumping. The energy inputs were divided into thermal energy (e.g., heating reactants, providing endothermic reaction heat) and electrical energy (e.g., for compressors, agitators, pumps) used within the fuel synthesis process. Many synthesis reactions are exothermic (Sabatier, methanol, FT polymerization, ammonia, HVO hydrotreating), which means that they release heat rather than consume it.^19^ In such cases, thermal energy may be needed only to start up or maintain the reactor temperature, after which the exothermic heat can often sustain operation (with cooling required to remove excess heat). If excess heat is high-quality, it could be reused elsewhere (in CO_2_ capture or for generating steam), potentially improving overall efficiency. In the case of biorefineries, waste streams can be used to produce the energy necessary for biobased processes. However, each processing block is treated as a stand-alone, and no cross-process heat or material integration credits are applied; this conservative baseline keeps pathway comparisons transparent.

Starting with literature data, the energy input table, in the Supporting Information, was constructed using the above data to compute, for each fuel, the following:

(1) Hydrogen energy input: broken into the portion that becomes chemical energy in fuel vs the portion lost as heat (this distinction will be reflected in the H_2_ use coefficient, but in input terms, all hydrogen energy comes from electricity via electrolysis). The electricity for H_2_ is listed as the input (since that is what is consumed from the grid).
(2) Feedstock energy input: for e-fuels, this is the energy to capture CO_2_ (thermal + electricity); for biofuels, this includes biomass LHV (since that is an energy input from nature).
(3) Synthesis process energy: any additional heat or electricity used in the fuel synthesis plant, not including the above.

## 3 Results

### 3.1 External-Energy Demand of the Investigated Pathways

Figure 2a condenses all primary data to show the total amount of external energy input required to deliver 1 MJ of chemical energy in the final product, and it presents these values as stacked horizontal bars where green equals the energy input for feedstock provision (CO_2_ capture for e-fuels; cultivation, harvesting, and pretreatment for bioroutes); the orange portion represents the energy input used inside the synthesis plant (reactor heating or cooling, compression, separation), and the light green bar shows the electricity consumed by the electrolyzer to supply the hydrogen requirement. Across the 11 pathways-to-fuel investigated, two clusters emerge. E-fuels synthesized exclusively from captured CO_2_ and green H_2_ display the highest demand, ranging from 1.81 MJ_H2_/MJ_Fuel_ for E-DME to 2.03 MJ_H2_/MJ_Fuel_ for long-chain FT hydrocarbons. Hydrogen electrolysis dominates the balance, confirming that the H/C-driven stoichiometric penalty predicted by eq 1 is paid as renewable electricity for electrolysis. OMEx retains oxygen in the molecule, so its light-green slice is the smallest for e-fuels (1.58 MJ_H2_/MJ_Fuel_), yet the total bar still requires 2.61 MJ_in_/MJ_Fuel_ overall because the oxygen retained in the fuel reduces its lower heating value (LHV < 20 MJ/kg). In contrast, bioderived routes consume as low as 0.07 MJ_H2_/MJ_Fuel_ for biojet/SAF; at the opposite, it exhibits the largest contribution from feedstock preparation (1.06 MJ_upgrading_/MJ_Fuel_): distillation and dehydration of dilute fermentation remain the dominant energy sinks despite the low hydrogen demand of the upgrading step (eq 4). For bioroutes, the G-H_2_ fraction never exceeds 1.38 MJ_H2_/MJ_Fuel_, as part of the hydrogen requirement is intrinsically supplied by the biomass. HVO/HEFA records the absolute minimum (0.19 MJ_in_/MJ_Fuel_) owing to the high intrinsic H/C ratio of triglycerides and the mild hydrotreating conditions documented in eq 3. Gasification-derived routes (BTL-FT, biomethane) sit midrange (1.52 MJ_in_/MJ_Fuel_), reflecting the trade-off between autothermal reforming, which provides process heat, and ex situ hydrogen addition to raise the H_2_/CO synthesis ratio. The light green bar tells us how much chemical energy from G-H_2_ production is embedded in each kilogram of fuel through the hydrogen-energy share metric HES% and the efficiency of the electrolyzer. For e-methane, the hydrogen-energy intensity HES is 60 MJ_H2_/MJ_Fuel_, which is 1.2 × the LHV of methane itself (approximately 50 MJ_CH4_/kg_CH4_). Hence, the hydrogen-energy share is HES% = 120%, contributing to a total external energy input for hydrogen of 200% (light green slice) when considering the efficiency of the electrolyzer. In other words, the hydrogen added during synthesis carries more chemical energy than the final fuel, a direct consequence of the CO_2_ + 4 H_2_ stoichiometry.

**Figure 2:**
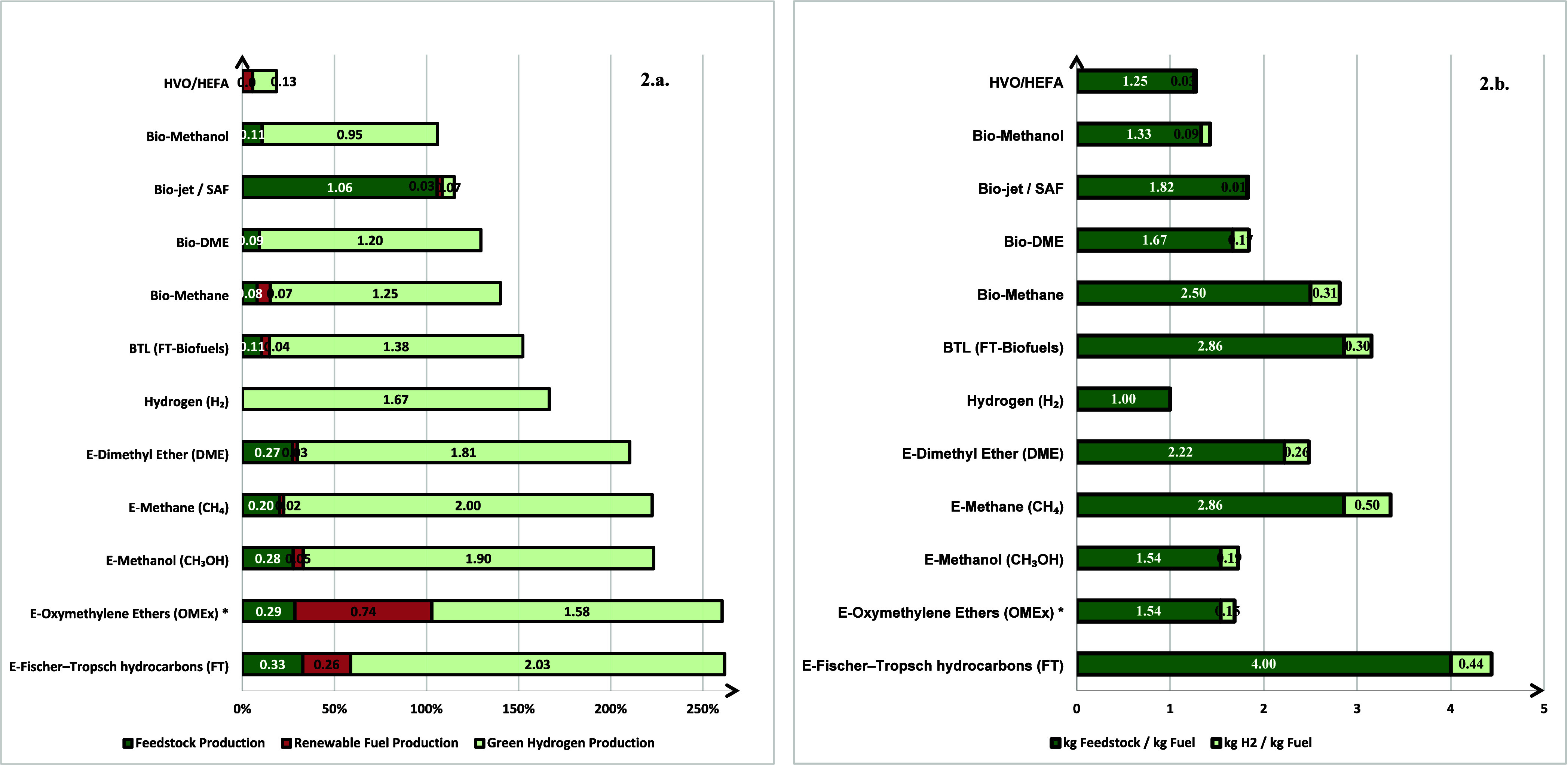
(a) Energy inputs normalized to the energy of the product fuel (MJ input per MJ fuel, 0 → 2.5 Mj/MJ, i.e., 0 → 250% of the fuel’s own LHV). Bars are split into feedstock-provision energy (dark green), in-plant utilities (orange), and green-hydrogen production (light green). The length of the light-green segment equals the hydrogen-energy share into the electrolyzer efficiency HES%ηel for each pathway. (b) Material inputs normalized to the mass of the product fuel (kg input per kg fuel). Bars are decomposed into feedstock provision (dark green) and green hydrogen supply *R*_H2_ (light green). Data sources and calculation details are given in the Supporting Information. *To calculate the hydrogen requirement a value of *n* equal to 10 was considered.

### 3.2 Material Inputs

Figure 2b expresses the same pathways on a mass basis. The quantity of external H_2_ scales with the green-energy penalty in Figure 2a, rising from 0.03 kg_H2_/kg_Fuel_ for HVO/HEFA to 0.50 kg_H2_/kg_Fuel_ for E-methane. Lipid hydrotreatment needs 1.25 kg_Biomass_/kg_Fuel_, whereas lignocellulosic syngas routes approach 3 kg_Feedstock_/kg_Fuel_ because of the lower LHV and higher ash/moisture content of wood. E-fuel pathways consume only captured CO_2_ (1.54–4.00 kg_CO2_/kg_Fuel_, depending on oxygen retention); this mass is subsequently released during combustion and therefore recycled in the global carbon balance.

### 3.3 Complementarity between E-Fuels and Biofuels

Figure 2 confirms the conceptual framework introduced before: electricity versus biomass leverage. E-fuels are electricity-intensive yet independent of biomass; on the contrary, biofuels conserve electricity but depend on limited biomass resources. The most electricity-efficient biofuel (HVO) consumes roughly one-tenth of the renewable power required by the most hydrogen-intensive e-fuel (E-FT). Hydrogen leverage is pathway specific: where renewable electricity is plentiful but sustainable biomass is scarce (e.g., offshore-wind regions, desert PV clusters), CO_2_-derived e-fuels provide a scalable option at the expense of high-power consumption. Conversely, in agricultural regions with limited grid decarbonisation, lipid- and lignocellulose-based routes deliver the greatest fuel yield per unit of electricity. Supplying 0.07 MJ of G-H_2_ upgrades 1 MJ of ethanol into drop-in SAF, whereas at least 2 MJ of G-H_2_ is needed to turn the same electricity into E-FT kerosene from CO_2_.

## 4 Discussion

For biofuels, the energy return is higher. The “free“ solar energy stored in biomass is abundant globally. It may seem counterintuitive to input more energy than is obtained in the fuel, as is the case for e-fuels where the thermodynamic cost is dominated by irreversible losses in electrolysis and CO_2_-hydrogenation reactors. Nevertheless, this higher process entropy generation trades with almost unlimited scalability with renewable electricity and available CO_2_, which could be captured from any industrial source or air. Thus, there is a compromise between efficiency and scalability: biofuels use less electrical energy per unit fuel (often reusing biomass energy with 30–50% conversion efficiency), while e-fuels can be produced with virtually no biomass but demand substantial electricity and therefore extensive renewable-capacity deployment.

The normalized metrics presented here provide a rapid and straightforward benchmark, simplifying the discussion of which pathway offers the greatest climate benefit per unit of electricity invested. When renewable power is cheap and plentiful (e.g., periods of oversupply), converting it into e-fuel can be economically sensible despite efficiency losses. E-fuels might thus become cost-competitive for aviation and shipping where alternatives (i.e., electrification of the sector) are limited. EU policy already sets distinct blending targets: the ReFuelEU Aviation Regulation, for example, requires 2% total SAF by 2025 rising to 70% by 2050, with a submandate of 1.2% e-SAF in 2030 and 35% e-SAF by 2050, the remainder expected from bio-SAF.^55^ Biofuels, although more efficient on an energy-input-to-output basis, face constraints related to feedstock collection, seasonal availability, and often higher feedstock cost unless waste residues are used. In regions where renewable electricity is scarce or expensive, policy should prioritize biofuels that deliver the greatest fuel output per MJ of power input; e-fuels become attractive where renewable-power oversupply or curtailment is common.^56^

## 5 Conclusions

Excess photovoltaic and wind electricity is already beyond battery capacity; electrolysis is the only scalable buffer, yet liquefying or compressing H_2_ forfeits more than 30% of its energy. Converting that H_2_ in situ to liquid fuels sidesteps the storage penalty, but the choice of fuel is nontrivial. The H_2_ intensity metrics introduced here resolve the trade-off in a single number.

Across 11 industrially relevant routes, external H_2_ inputs were found to span 2 orders of magnitude: from 0.07 MJ_H_2__/MJ_Fuel_ for ethanol-to-jet and 0.19 MJ_H_2__/MJ_Fuel_ for lipid-derived HVO, up to 2.62 MJ_H_2__/MJ_Fuel_ for e-methane. Bioroutes therefore can deliver roughly three times more liquid-fuel energy per kilowatt-hour of renewable electricity than e-fuels, but their scale is bounded by sustainable biomass. Conversely, e-fuels consume 1.6–2.0 MJ_H_2__/MJ_Fuel_ yet can expand almost without limit wherever cheap power and concentrated CO_2_ coexist. The metric thus prescribes a two-tier deployment strategy: prioritize low-HES biofuels where biomass is available; channel curtailed renewables into high-HES power-to-liquid fuels where it is not. Aviation typifies the latter case, where European biomass can meet <70% of the projected jet demand, while ReFuelEU sets a 35% e-SAF mandate by 2050. Directing surplus solar and wind electricity into synthetic SAF (HES% more than 120%) simultaneously solves long-duration storage and decarbonizes the hardest-to-electrify sector. While the present analysis is intentionally limited to hydrogen-energy metrics, real deployment decisions must also consider costs, technology maturity, scale-up potential, and infrastructure; combining HES/HES% with detailed techno-economic and environmental assessments is therefore a natural next step for holistic comparisons. Liquid hydrogen storage vectors such as ammonia and liquid organic hydrogen carriers (e.g., cyclohexane–benzene) are important competing options to fuel production pathways, acting as “hydrogen tanks” that enable flexible storage and release of green hydrogen when surplus renewable electricity is available. Beyond the liquid fuel pathways considered here, the HES/HES% framework could in principle be extended to such storage vectors by treating the storage–release chain as an additional pathway and redefining the underlying hydrogen and feedstock terms where suitable data and process models are available.

## Supplementary Material



## Notes

Table S1 - metrics: Inputs and calculated hydrogen-intensity metrics (η_el_, RH_2_, HES, HES%) and energy/mass inputs by pathway; Table S2 - H_2_ data: Hydrogen production routes with TRL and indicative energy requirements; Table S3 - CO_2_ data: CO_2_ sourcing/capture routes with TRL and indicative energy requirements (XLSX)

## GLOSSARY

Biofuel: Fuel from biomass
Bio-DME: Biomass-derived dimethyl ether
Biojet/SAF: Biomass-derived sustainable aviation fuel
Bio-Methane: Biomass-derived methane
Bio-Methanol: Biomass-derived methanol
BTL: Biomass-to-Liquid hydrocarbons
BTX: Benzene–toluene–xylenes
Drop-in fuel: Direct fossil substitute
E-DME: Dimethyl ether from CO_2_ + G-H_2_
E-FT: Fischer–Tropsch hydrocarbons from CO_2_ + G-H_2_
E-fuel: Fuel from CO_2_ + G-H_2_
E-Methane: Methane from CO_2_ + G-H_2_
E-Methanol: Methanol from CO_2_ + G-H_2_
E-OMEx: Oxymethylene ethers from CO_2_ + G-H_2_
**G-H**_**2**_: Green hydrogen
**H**_**2**_: Molecular hydrogen
HES: Hydrogen-Energy Share coefficient
HES %: Hydrogen Energy fraction
HVO/HEFA: Hydrotreated vegetable oil/hydroprocessed esters and fatty acids
kWh: Kilowatt-hour
LHV: Lower heating value
LPG: Liquefied petroleum gas
MJ: Megajoule
PtX: Power-to-X (surplus renewable electricity into chemical products)
**R**_**H2**_: Specific hydrogen demand
FT: Fischer–Tropsch
VRES: Variable renewable energy sources
WGS: Water–gas shift reaction
